# Atypical enteropathogenic *Escherichia coli* associated with an acute diarrhoeal disease outbreak due to fermented soybean in Keifang, Mizoram, India: a challenge toward food safety and prevention efforts

**DOI:** 10.3389/fpubh.2026.1722805

**Published:** 2026-04-01

**Authors:** Swagnik Roy, Rebecca Lalngaihzuali, Samaresh Das, Shalony Roy, Parimal Roychoudhury, Fatema Akter, Gracy Laldinmawii, Jacinta Lalmunsangi, T. Ramamurthy, Ashis Kumar Mukhopadhyay, Madhuchhanda Das, Tapan Kumar Dutta

**Affiliations:** 1 Zoram Medical College, Aizawl, Mizoram, India; 2 Centre for Development of Advanced Computing (CDAC), Kolkata, West Bengal, India; 3 CVSc&AH, Central Agricultural University, Aizawl, Mizoram, India; 4 ICMR-National Institute for Reserach in Bacterial Infections (NIRBI), Kolkata, West Bengal, India; 5 Indian Council of Medical Research, New Delhi, India

**Keywords:** acute gastroenteritis, aEPEC, fermented soybean, India, Mizoram

## Abstract

**Introduction:**

Foodborne and waterborne gastrointestinal disease outbreaks are frequently reported from the northeastern region of India.

**Methods:**

In the present study, an outbreak of acute gastroenteritis in Keifang village of Mizoram, India, was investigated following official notification through the state Integrated Disease Surveillance Program (IDSP) unit.

**Results:**

The investigation involved descriptive epidemiology and exposure assessment for 40 affected individuals. Stool samples from all affected patients, healthy control individuals (*n* = 20), and leftover food specimens (*n* = 2) were collected for laboratory analysis. All samples were processed using standard microbiological methods, conventional PCR, and real-time PCR assay for the identification of causative agents. Pure bacterial cultures underwent antimicrobial sensitivity testing. Data were analyzed to determine the attack rate, construct the epicurve, and perform spot mapping of the outbreak. Atypical enteropatho genic *Escherichia coli* (aEPEC) was isolated from all 40 affected individuals and one food specimen. No other significant microbial pathogens, including bacteria, viruses, or parasites, could be detected in the specimens by any method.

**Discussion:**

All the aEPEC isolates were sensitive to most antimicrobials, except ampicillin and ceftazidime. Since all the infected patients consumed the same fermented soybean purchased from a single vendor, and aEPEC was isolated from both the patients and the fermented soybean, this suggests a strong correlation between the aEPEC isolates and the outbreak in Keifang, Mizoram, India, which was transmitted through fermented soybean.

## 1 Introduction

Food poisoning is a common but often underestimated health issue that affects millions of people worldwide each year. According to the World Health Organization (WHO), 600 million people fall ill annually, and 420,000 die as a result of consuming unsafe food. Among these, the WHO South-East Asia Region reports the highest number of cases, with 150 million people affected and 175,000 deaths annually.^1^

Soybean is considered a valuable and cheap source of protein, and accordingly, a high demand for soybean arises in the community, particularly among the lower-income group people in the northeastern region of India. In this region, soybean is commonly consumed in the form of fermented products, such as bekang in Mizoram, India. Fermentation of soybean generally involves the breakdown of complex substrates through the action of microorganisms, such as molds, yeasts, and bacteria, to either generate desirable flavors or act as a preservation technique (1). However, many studies reported that consumption of spontaneously fermented soybean products, which is a local cuisine in this region, may cause various health issues due to the presence of several potential hazards, such as bacterial pathogens, mycotoxins, and biogenic amines (BAs), which can lead to health problems (2). Rai et al. (3) studied the traditional fermented soybean product in Nepal and isolated *Escherichia coli*, *Salmonella enteritidis*, *Shigella sonnei*, *Staphylococcus aureus,* and various other pathogenic bacteria. Due to the lack of strict safety guidelines for the preparation of fermented soybean products, traditional fermentation often uses spontaneous inoculation to initiate the fermentation process (4), which poses a risk of the introduction of microorganisms that have the ability to produce toxins (5).

Diarrheagenic *Escherichia coli* (DEC) is one of the major causes of foodborne gastroenteritis in human beings. Among the DEC, enteropathogenic *E. coli* (EPEC) is the first described pathotype. EPEC is the leading cause of infantile diarrhea, particularly in developing countries, where hygiene quality is compromised (6). EPEC pathotypes produce an outer membrane protein called intimin, through which they can bind host cell intestinal epithelium very tightly and develop attaching and effacing lesions. The intimin protein is encoded by the *eaeA* gene of EPEC pathotypes (7). Bundle-forming pili, encoded by the *bfpA* gene, are another important, but not essential, virulence factor of EPEC, which helps with the initial attachment of the bacteria. The EPEC isolates positive for *eaeA* and *bfpA* genes are classified as typical EPEC (tEPEC), and those positive for the *eaeA* gene but negative for the *bfpA* gene are classified as atypical EPEC (aEPEC) (8). Although tEPEC are widely reported as the major causative agent of acute gastroenteritis, there are a handful of reports on the association of aEPEC as the primary cause of acute gastroenteritis (6).

In the present study, an acute outbreak of gastroenteritis among the population of Keifang village, Saitual district of Mizoram, India, was investigated for laboratory confirmation of the causative agent. Upon investigation, aEPEC, which may have been transmitted through fermented soybeans (Bekang), was found to be strongly associated with the outbreak.

## 2 Materials and methods

### 2.1 Outbreak setting

Upon the notification of an unusual occurrence of acute gastroenteritis at Police Veng, Keifang, Saitual district of Mizoram, on 11 September 2024, a team from Zoram Medical College, Aizawl, Mizoram, initiated the investigation. A total of 40 people out of 58 from 10 families showed mixed symptoms of fever, diarrhea/ loose stool, abdominal pain, vomiting, and headache. The majority of them exhibited symptoms after having dinner on 7 September 2024, and a few of them showed symptoms after breakfast on 8 September 2024, at their home. A total of 18 individuals from 10 families did not show any symptoms. A total of 10 individuals required hospitalization and were admitted to the nearest district hospital at Saitual, Mizoram.

The common ingredient in the food items consumed was found to be fermented soybean product (Bekang), a local condiment of the Mizo cuisine. All 10 families purchased the fermented soybean from a local woman, who used to ferment the soybeans by herself regularly. Raw soybean was procured from a nearby village, Buhban, approximately 25 km from Keifang, during March 2024 and was stored at her home unrefrigerated for 6 months before fermentation. After the fermentation process, the product was wrapped in fresh tree leaves, which were collected from the forest and used unwashed, and finally sold at the roadside market.

### 2.2 Case definition

A case is defined as an individual suffering from acute watery diarrhea in the village who presented with abdominal pain, vomiting, and/or diarrhea from 7 to 11 September 2024. Acute watery diarrhea is defined as the passage of loose and liquid stool more than three times a day.

### 2.3 Case study

The outbreak was analyzed based on descriptive epidemiology and exposure assessment for 40 affected individuals, along with 20 control individuals. The history and details about the symptoms were collected from all the individuals through personal interviews.

### 2.4 Case control selection criteria

Individuals (*n* = 40) suffering from acute watery diarrhea in the village and presenting with abdominal pain, vomiting, and/or diarrhea from 7 to 11 September 2024 were considered affected, and samples were collected from them. Individuals (*n* = 20) without any history of abdominal pain, vomiting, and/or diarrhea from 7 to 11 September 2024 and without any history of consumption of fermented soybeans were considered controls, and stool samples were collected from them.

### 2.5 Sample collection and transportation

Stool samples were collected from all the affected individuals (*n* = 40) in a sterile container containing Stuart’s Transport medium and carefully transported to the laboratory within 2 h under cold chain. Stool samples were also collected from 20 healthy individuals residing at Police Veng, Keifang, as controls. Two leftover food items (fermented soybean), which were consumed by all the members of the 10 affected families, were also recovered from the area and collected for analysis. One food sample (raw soybean seeds), which was known to be the raw material for preparing fermented soybeans responsible for the outbreak, was also recovered and collected for further analysis.

### 2.6 Sample processing and laboratory testing

Upon receiving, all the stool samples were initially examined by the unaided eye for consistency, presence of blood, or any helminths. A wet mount of the stool specimens was also prepared for the detection of parasitic ova or cysts.

For bacteriological analysis, all the samples were processed for isolation and identification of possible bacterial pathogens following the methods described in the Standard Operating Procedures, ICMR Foodborne Pathogens Survey and Research Network (NE Region), 2024. In brief, all the samples were inoculated in two different processes: direct inoculation on selective agars such as MacConkey’s agar, deoxycholate citrate agar (DCA)/xylose lysine deoxycholate (XLD), and thiosulfate citrate bile salt sucrose (TCBS) agar (HiMedia, Mumbai) followed by incubation under an aerobic environment at 37 °C for 18–24 h; another batch was inoculated in enrichment broth and incubated aerobically at 37 °C for 18–24 h. In case of any turbidity in the enrichment broth, the culture was re-inoculated in suitable selective media for further incubation at 37 °C for 18–24 h. All the selected pure bacterial colonies were further studied following standard bacteriological techniques and confirmed by the VITEK2 automated bacterial identification system (BioMérieux, France), as well as *E. coli* pathotypes (EPEC, EAEC, STEC/EHEC, EIEC, ETEC, and DAEC) specific PCR assays using specific oligonucleotide primers (Table 1) (ICMR Foodnet SOP, 2024). All the bacterial isolates were further verified and confirmed by the ICMR National Institute for Research in Bacterial Infections (NIRBI), Kolkata, India, which is an External Quality Assurance Service (EQAS) center. All the isolates were confirmed based on a battery of microbiological, serological, and molecular assays.

**Table 1:** Details of oligonucleotide primers used for the determination of the *E. coli* pathotypes.

Sl No	Name of the pathotype	Name of the target gene	Primer sequence (5′-3′)	Expected amplicon size (bp)	Reference
1	ETEC	*elt*	F: CACACGGAGCTCCTCAGTC R: CCCCCAGCCTAGCTTAGTTT	508	(26)
		*est*	F: GCTAAACCAGTAG/AGGTCTTCAAAA R: CCCGGTACAG/AGCAGGATTACAACA	147	
2	EPEC	*eaeA*	F: CCCGAATTCGGCACAAGCATAAGC R: CCCGGATCCGTCTCGCCAGTATTCG	881	(26)
		*bfpA*	F: GGAAGTCAAATTCATGGGGG R: GGAATCAGACGCAGACTGGT	367	
3	EAEC	*aatA*	F: CTGGCGAAAGACTGTATCAT R: CAATGTATAGAAATCCGCTGTT	630	(26)
		*aaiC*	F: ATTGTCCTCAGGCATTTCAC R: ACGACACCCCTGATAAACAA	215	
4	EHEC	*stx1*	F: CAGTTAATGTGGTGGCGAAGG R: CACCAGACAATGTAACCGCTG	348	(27)
		*stx2*	F: ATCCTATTCCCGGGAGTTTACG R: GCGTCATCGTATACACAGGAGC	584	
5	EIEC	*ipaH*	F: CTCGGCACGTTTTAATAGTCTGG R: GTGGAGAGCTGAAGTTTCTCTGC	933	(27)
6	DAEC	*daaE*	F: GAACGTTGGTTAATGTGGGGTAA R: TATTCACCGGTCGGTTATCAGT	542	(27)

All the specimens were also processed for the presence of diarrheogenic viral pathogens (*Rotavirus*, *Adenovirus*, *Norovirus* genotype I and II, *Sapovirus,* and *Astrovirus*) following the same standard operating procedures and using the real-time PCR multiple viral pathogen detection kit (GCP-Q Comprehensive Real Time PCR kit for Gastro-intestinal Comprehensive Panel) following the guidelines mentioned by the manufacturers. The same kit was also used for the detection of common diarrheagenic parasites, including *Giardia lamblia*, *Entamoeba histolytica*, *Cryptosporidium* spp., *Cyclospora cayetanensis*, and *Dientamoeba fragilis*.

All the bacterial isolates were further analyzed for their antimicrobial resistance profile by disk diffusion assay using specific antimicrobial disks (ampicillin, cefepime, cefotaxime, cefoxitin, ceftazidime, chloramphenicol, ciprofloxacin, erythromycin, gentamicin, imipenem, nalidixic acid, tetracycline, and trimethoprim-sulfamethaxozole) (HiMedia, Mumbai, India) or broth dilution assay using specific antibiotic salts (HiMedia, Mumbai, India) as per the guidelines of CLSI (2024) (9).

All the food samples, including fermented soybean and raw soybean, were also processed for recovery of any microbial pathogens following the procedure of ICMR Foodnet SOP (2024).

### 2.7 Data analysis

The data analysis was performed with Microsoft Excel, Office 365 version, to calculate the attack rate of the population by age and gender. The entire outbreak data were uploaded to the ICMR-FoodNet web portal for automated data analysis, including determination of attack rates, development of the epicurve, generation of a spot map, and other descriptive epidemiological analyses.

## 3 Results

### 3.1 Descriptive epidemiology

**Figure 1:**
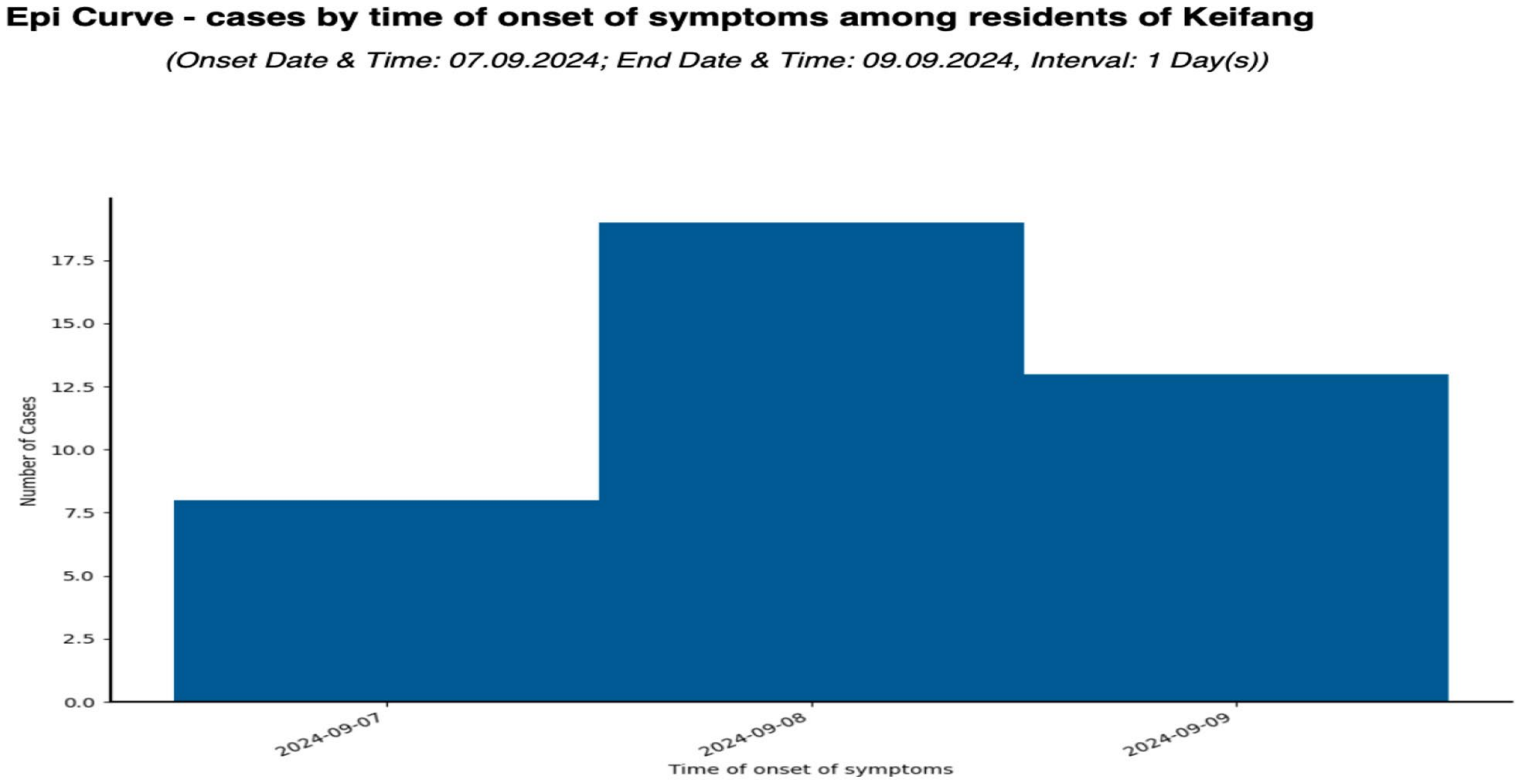
Epidemiological curve of the acute gastroenteritis outbreak due to aEPEC at Keifang, Mizoram, India, indicating the date of onset and end date of the outbreak and its total duration.

**Figure 2:**
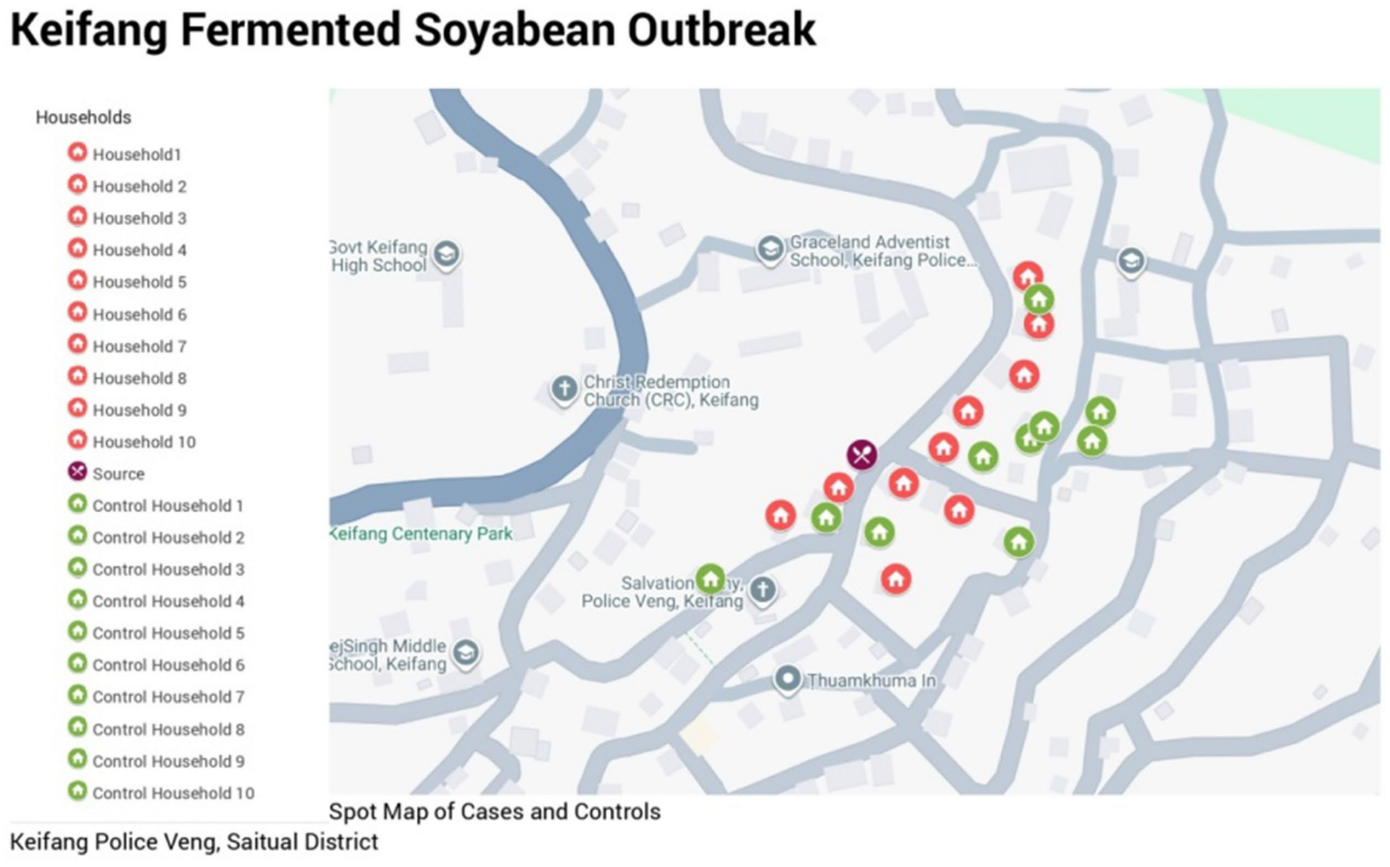
Spot map of the acute gastroenteritis outbreak due to aEPEC at Keifang, Mizoram, India, depicts the affected households (red), control households (green), and source of infection (purple).

The total population of the affected village is 4,539, where the female population is higher than the male (1,012 females vs. 1,000 males) (Figure 1). A total of 40 affected cases were interviewed, and a detailed line listing was compiled. Analysis of the epidemic curve (Figure 2) showed that similar symptoms were recorded over 3 consecutive days, from 7 to 9 September 2024. The first onset of symptoms occurred after consumption of food items containing fermented soybean on 7 September between 3:00 and 10:00 p.m. among eight people aged 12–75 years. The second onset of symptoms occurred on 8 September between 5:00 a.m. and 6:15 p.m., among 19 people aged 20–45 years, and the third onset occurred on 9 September between 2:00 a.m.and 4:00 p.m. among 13 people aged 4–72 years. All the observations indicated that the cases originated from the consumption of common food or water source. This hypothesis was further supported after interviewing all the affected individuals, all of whom had consumed fermented soybean procured from a single source.

Out of 40 affected cases, 10 were hospitalized but recovered after treatment with suitable antibiotics and fluid therapy. The remaining cases were self-limiting and recovered within 24 h after the onset of symptoms. The attack rate (87.5%) was highest among the older adult group (≥61 years), whereas children up to 5 years showed the lowest attack rate (50%) (Table 2). Gender-wise, the attack rate was higher among females (43.10%) compared to males (25.86%) (Table 3). The higher attack rate among older adults, particularly females, further supported fermented soybeans as the likely source, as this product is more commonly consumed by older women compared to men and younger kids.

**Table 2:** Attack rate of various age groups.

Age group (years)		Population interviewed (*n* = 58)	Number of cases (*n* = 40)	Attack rate (%)
0–5	2	1	50	
6–20	15	12	80	
21–40	22	13	59.1	
41–60	11	7	63.63	
61 and above	8	7	87.5	

**Table 3:** Attack rate by sex.

Sex	Population	No. of cases	Attack rate (%)
Female	58	25	43.10
Male	58	15	25.86
Overall	58	40	68.96

### 3.2 Laboratory results

Upon analysis, atypical enteropathogenic *Escherichia coli* (aEPEC) was isolated and identified from all 40 affected individuals and one of the fermented soybean (Bekang) samples recovered from the vendor. All the isolates were found to be positive for the intimin gene (*eaeA*), but none were positive for bundle-forming pili (*bfpA*) gene, which indicates that all the isolates were atypical EPEC. In addition, non-diarrheagenic *E. coli* and non-toxic *Bacillus cereus* were also recovered from the feces of affected patients. EPEC was not recovered from the feces of any of the healthy controls. In addition, based on the results of the GCP-Q comprehensive real-time PCR assay, all the samples were positive for EPEC and negative for *Shigella* spp., *Salmonella* spp., *Yersinia enterocolitica*, *Campylobacter* spp., enterotoxigenic *E. coli* (ETEC), enteroaggregative *E. coli* (EAEC), verotoxigenic *E. coli* (VTEC/STEC), enteroinvasive *E. coli* (EIEC), diffusely adherent *E. coli* (DAEC), *Vibrio* spp., *Clostridium difficile* Toxin A, *Clostridium difficile* Toxin B, *Helicobacter pylori,* and *Staphylococcus aureus*.

Although there were a few common parasitic ova detected in the feces of 7 of the 40 affected individuals, no diarrheagenic viruses or parasites could be detected by the real-time PCR assay. All laboratory observations indicated that the present outbreak was due to consumption of fermented soybean contaminated with aEPEC. The same fermented soybean might be contaminated by water sources.

All the aEPEC isolates were fairly sensitive to most of the antibiotics except ampicillin and ceftazidime. It is important to mention that most of the hospitalized patients recovered due to timely treatment with suitable antimicrobials selected through the AST result of the study.

## 4 Discussion

Food poisoning, a common yet preventable public health issue, remains a significant concern worldwide. It results from consuming food contaminated with pathogenic microorganisms or their toxins. Among the many pathogens responsible for food-borne illnesses, enteropathogenic *Escherichia coli* (EPEC) is notable for its ability to cause gastrointestinal distress, especially in home environments, where food safety practices may be inconsistent.

In the present study, aEPEC was found to be associated with the acute gastroenteritis outbreak among the 40 people in Keifang village of Mizoram, India, due to consumption of contaminated fermented soybeans (Bekang). Acute diarrheal disease (ADD) outbreaks are commonly reported phenomena in the Eastern region, particularly the Northeastern region of India. A major contributing factor is the lack of awareness among the population and an inadequate healthcare system to control acute outbreaks (10). Recently, an outbreak of waterborne acute diarrheal disease was reported in a South Tripura district, India, due to the consumption of contaminated water (10). In a study, it was observed that irrespective of pathogen, the leading cause of food poisoning outbreaks is improper food handling and preparation, accounting for 93% of cases; notably, 16% of these take place in households or private residences (11, 12). The reported mishandling practices included leaving food at room or outdoor temperatures for extended periods (58%), inadequate reheating in terms of time or temperature (57%), slow cooling of cooked items (44%), undercooking during the initial preparation (40%), preparing meals more than 12 h before serving (33%), improper hot holding with insufficient heat or duration (33%), and failing to maintain proper cold storage temperatures (22%). The most frequently cited issues included improper cleaning of equipment or utensils (67%) and storing food in contaminated environments (39%) (11, 12). The food poisoning outbreak due to EPEC was also reported from Maharashtra, India, where approximately 4,000 people were affected due to the consumption of lunch at religious gatherings, where food was prepared under temporary arrangements, and the source of the outbreak was not identified due to the non-availability of the food items (13).

The pathogenicity of typical EPEC (tEPEC) is well known and has been confirmed by volunteer studies and epidemiological studies, whereas the role of aEPEC as the major cause of human diarrhea remains controversial (14). In a study (15), it is reported that the aEPEC O128:H2 strain could not develop diarrhea in adult volunteers. Similarly, in another volunteer study, a tEPEC O127:H6 strain without the EAF plasmid was found to be less virulent and justified the role of bfp in the development of diarrhea (16). Few epidemiological studies highlighted the association of aEPEC with diarrhea, whereas few other reports indicated no difference in the isolation ratio of aEPEC between diarrheal patients and controls (17). It is important to note that numerous reports of outbreaks associated with aEPEC have established and confirmed the role of this pathogen as a diarrheagenic agent. In the year 1987 in Finland, a large outbreak, which involved 611 students and 39 adults, was associated with aEPEC O111:B4 strain (18). In 1991, another large foodborne outbreak caused by aEPEC O39:HNM strain involved more than 100 patients (19). A waterborne outbreak caused by aEPEC ONT:H45 was also reported in Japan (20). Similarly, aEPEC O157:H45 and O127a:K63 were also reported as the causative agents of foodborne outbreaks in 2013 (21) and 2010 (22). Recently, in June 2022, a large foodborne outbreak occurred following the consumption of a contaminated boxed lunch in the Kinki region of Japan, and upon investigation, aEPEC O45:H15 was recorded as an etiological agent of the outbreak (6). All these findings, including our present study, suggest that some, but not all, aEPEC strains are virulent to humans, although the genetic background of these outbreak-related strains is poorly understood (22, 23).

The present outbreak was linked to the consumption of fermented soybean (Bekang), which is a local cuisine among the ethnic Mizo population. As mentioned in the earlier section, the method of preparation of the said product is always questionable due to improper hygienic practices. The raw materials and the utensils are not maintained properly to prevent any bacterial contamination. The products used to cover in tree leaves, which are collected from the forest and might be washed in the stream, which carries many diarrheagenic bacteria. The distant areas of the state do not have any source of treated water, and the entire population is dependent on stream water. On many occasions, the common water source is contaminated with enteric bacteria through animal feces (24, 25). The vendor used to prepare the same product regularly and sell it at the weekly market. aEPEC was also recovered from the fermented soybean product, which may have been contaminated via water or the tree leaves used for packaging. A definitive conclusion regarding the exact source of contamination could not be determined due to the unavailability of water and the lack of samples from the same vendor.

Detection of pathogenic bacteria from locally prepared food items indicates the necessity of routine surveillance and intervention programs at the village-level markets to maintain food safety. It is important to note that after the swift scientific intervention through the state Integrated Disease Surveillance Program (IDSP), the food vendors started following hygienic practices, and no more outbreaks have been reported so far.

### 4.1 Limitations of the study

The food handler’s kitchen at her home had already been cleaned when the investigating team reached the place. Therefore, a sample could not be collected from the kitchen workplace. The other food items consumed along with the fermented soybean for dinner and breakfast, as well as the leaves used for wrapping the soybean during fermentation, were also discarded before collection. In addition, we could not perform strain-level typing or whole-genome sequencing of the isolates to establish a definitive conclusion. The study was limited by potential recall bias and a small sample size.

## 5 Conclusion

Atypical EPEC was found to be strongly associated with acute gastroenteritis in a remote village of Mizoram, India, due to the consumption of contaminated fermented soybeans. Although aEPEC is not considered a major pathogen causing gastroenteritis in humans, this outbreak report provides an opportunity to re-examine the role of the organism. Furthermore, the report underscores the need for local authorities to develop suitable strategies to curb such incidents in the future.

## Data Availability

The datasets presented in this study can be found in online repositories. The names of the repository/repositories and accession number(s) can be found in the article/supplementary material.

## Footnotes

1
https://www.who.int/data/gho/data/themes/who-estimates-of-the-global-burden-of-foodborne-diseases
